# Using a Single-Screw Cortical Disc for a Simplified Shell Technique: A Novel Approach and Technical Note

**DOI:** 10.7759/cureus.86475

**Published:** 2025-06-21

**Authors:** Mehdi Ekhlasmandkermani, Fatemeh Goudarzimoghaddam, Mohammad Sabet Jahromi, Emir Ilkerli

**Affiliations:** 1 Dentistry, School of Dentistry, Kerman University of Medical Sciences, Kerman, IRN; 2 Clinical Research Development Unit, Mehrgan Hospital, Kerman University of Medical Sciences, Kerman, IRN; 3 Department of Periodontics, School of Dentistry, Babol University of Medical Sciences, Babol, IRN; 4 Department of Orthopedics and Traumatology, OsteMed Klinik Bremervörde, Bremervörde, DEU

**Keywords:** allograft, alveolar ridge augmentation, bone graft, cortical bone plate, dental implants, shell technique

## Abstract

Background

One of the notable challenges in implant dentistry is the inadequacy of bone dimensions. While guided bone regeneration remains the standard approach for horizontal bone reconstruction, emerging new approaches such as the shell technique offer the potential for significant bone gain through effective space creation. This article presents a modified shell technique as a promising and practitioner-friendly solution for managing horizontal bone deficiencies before and during implant placement. This article aims to introduce a simplified and minimally invasive approach that enhances space maintenance and surgical management and allows for extraoral screw fixation to the disc with just a single screw.

Methodology

This study included six patients with horizontal bone defects that made them candidates for bone augmentation procedures. We employed a newly modified shell technique utilizing a single-screw cortical disc. The cortical discs were fabricated from cortical plates using a trephine drill and Cortico-Cage device. A titanium screw was secured extraorally to the cortical disc. The assembly of the screw and disc was anchored bi-cortically in the appropriate position, and the gap between the cortical disc and the underlying bone bed was filled with a combination of autogenous chips and allograft material. This simplified shell technique is designed to facilitate bone augmentation in areas with horizontal bone deficiencies.

Results

Cone-beam computed tomography assessments performed five months postoperatively in five cases demonstrated that the increase in bone width at the crestal area, specifically at the site of cortical discs, ranged from a minimum of 2.03 mm to a maximum of 5.76 mm. Moreover, the initial bone width before reconstruction in the evaluated cases ranged from a minimum of 1.74 mm to a maximum of 4.40 mm. Radiographs before and after the procedure indicated a noteworthy bone formation.

Conclusions

The findings suggest that employing a single screw to secure the cortical disc while connecting it to the disc outside of the patient’s oral cavity can facilitate the surgical process and enhance patient comfort.

## Introduction

The bone resorption occurring after tooth removal depends on factors such as trauma, periodontal pathology, endodontic infection, and general health conditions [1], which represents a significant challenge in dental implant treatment. This resorption can manifest in horizontal and vertical dimensions, potentially leading to inadequate bone volume in the recipient area. These deficiencies make the implant’s proper prosthetic placement more difficult, jeopardizing the attainment of the ideal functional and esthetic outcomes [2]. Research has established that a minimum of 1.5 mm buccal bone width around dental implants is necessary to ensure long-term success [3]. In areas where such a minimum value is unattainable, bone regeneration techniques to provide an adequate implant foundation are indicated.

Over the past several decades, numerous approaches have been devised to increase bone volume tailored to the individual patient’s specific situation, the extent of the bone resorption, and the particular treatment goals. These techniques enable the reconstruction of bony defects and create an optimal bone bed for future dental implants. The most widely employed horizontal bone regeneration techniques include guided bone regeneration (GBR), ridge splitting, block grafting, and distraction osteogenesis [1,4-7].

Among the numerous techniques proposed, GBR has been recognized as a more conservative and straightforward method for mild-to-moderate reconstruction. This technique is characterized by a shorter reconstruction time and successfully maintains the space necessary for the new bone formation by using both resorbable and non-resorbable membranes. Nevertheless, a notable challenge with GBR is the progressive loss of bone volumetric stability over time and the risk of the resorbable membranes collapsing. These aspects can negatively affect the amount and quality of the newly formed bone [8].

Successful reconstruction relies heavily on the graft material’s stability and the efficacious mitigation of stress in the grafted zone. These parameters are critical in maintaining the integrity of the blood clot and ultimately contribute significantly to the overall success of the reconstruction [9,10]. In this context, one promising method for bone regeneration involves using bone plates as rigid barriers [11] to maintain or create space for effective bone regeneration. A recognized technique developed by Khoury employs autogenous bone plate [12]. The bone plates employed in this method can be sourced from three primary categories, namely, autogenous [13], allograft [14], or xenograft [15]. This approach, commonly called the shell technique, facilitates the reconstruction of various sizes of bony defects.

When selecting the shell technique for surgical operation, a series of significant factors must be assessed, including the defect’s type, size, morphology, and the quality of surrounding soft tissue. The implementation of rigid barriers in the shell technique offers a significant advantage. It promotes sustaining a stable space in vertical and horizontal directions, especially for large defects [16]. However, this technique presents challenges due to the intricate nature of the surgical procedure, which requires a high level of expertise in both shell fixation and soft tissue management to mitigate the risk of early exposure of the bone plate [17].

The original method of allograft plate, which is called the shell technique, typically involves a prefabricated rectangular plate, which is sometimes trimmed to adapt to the individual morphology of a defect, frequently necessitating several screws for fixation. In this article, we present a novel allograft plate design characterized by its cortical disc shape, which is produced with a trephine bur and can be secured with just a single screw. This technique represents a simplified and minimally invasive modification that facilitates space maintenance and surgical handling and enables extraoral screw fixation to the cortical disc, making it a promising option for clinical application.

## Materials and methods

**Figure 1:**
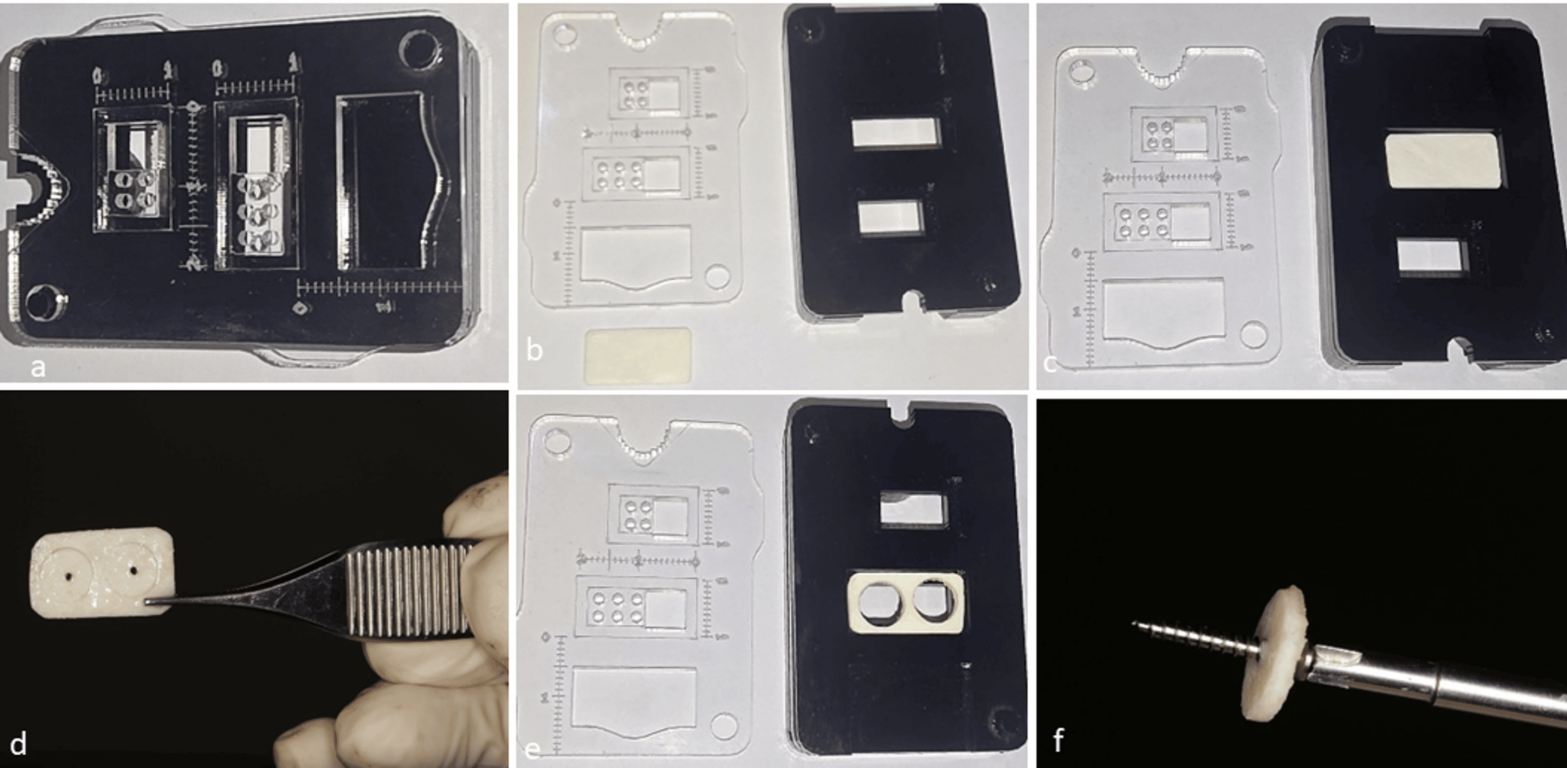
Overview of the Cortico-Cage: structure and applications. (a) The Cortico-Cage device is designed to prepare cortical discs. (b) Cortico-Cage comprises two primary components, i.e., a body and a cap. The cap features designated chambers for drill insertion and trephine burs, facilitating the preparation of allograft plates. (c) The desired dimensions of the cortical plate are positioned within the body’s chamber. (d) One or more cortical discs are cut based on the selected plate size. Before cutting the cortical disc with the trephine, central holes are created to facilitate the preparation of the hole. (e) Following the preparation of the central hole, cutting proceeds with the trephine bur until the discs are fully separated from the cortical plate. (f) After the cortical disc is fully separated, a screw with appropriate length is secured into the cortical disc outside of the patient’s oral cavity.

This study presents six successful cases of bone regeneration utilizing the simplified shell technique. All cases exhibited horizontal alveolar bone resorption, necessitating bone regeneration before or during implant placement. The treatments were performed in a private practice setting by a single operator between 2023 and 2025. Written informed consent was obtained from all six patients after a full explanation of the surgical procedure. In each instance, one or more cortical allograft plates (Iranian Tissue Product Company) with a customized thickness ranging from 1.2 to 1.4 mm were immersed in sterile saline for a minimum of 20 minutes to ensure optimal rehydration [14]. Then, utilizing a trephine bur (Juya Elekteronik; Iran) with an internal diameter of 8 mm, one or more cortical discs were meticulously excised from the cortical allograft plates. A specialized device known as the Cortico-Cage (International Classification: A61B 17/17; A61B 17/80; A61C 19/00) has been developed and registered to prevent the movement of the cortical plate during trephine bur use. The Cortico-Cage consists of two main components, namely, the body and the cap. The internal structure of the body is specifically designed to accommodate prefabricated cortical plates (Figures 1a, 1b). To initiate the procedure, the cortical plate of the appropriate size is positioned within the designated chamber, and then the exact location of the disc is accurately determined using a trephine bur (Figure 1c). Before the complete removal of the disc, a central screw hole is created, which serves to stabilize the disc and prevent any rotation during the drilling process (Figure 1d). Subsequently, the perforated disc is entirely detached from the plate through the continued use of the trephine bur (Figure 1e). The number of cortical discs required is determined based on the dimensions of the bone defect. Subsequently, a hole with a diameter of 1.4 mm was created at the center of each cortical disc. A self-tapping titanium screw (1.6 mm) was then placed extraorally into the cortical disc to ensure secure integration (Figure 1f). The diameter of the screw and the corresponding hole should be designed to ensure that the screw achieves optimal engagement with the cortical plate. This is essential for minimizing any cortical disc movement during the healing process. Additionally, the length of the screw should be determined based on the necessary space required between the disc and the defect bed.

## Results

Case 1

**Figure 2:**
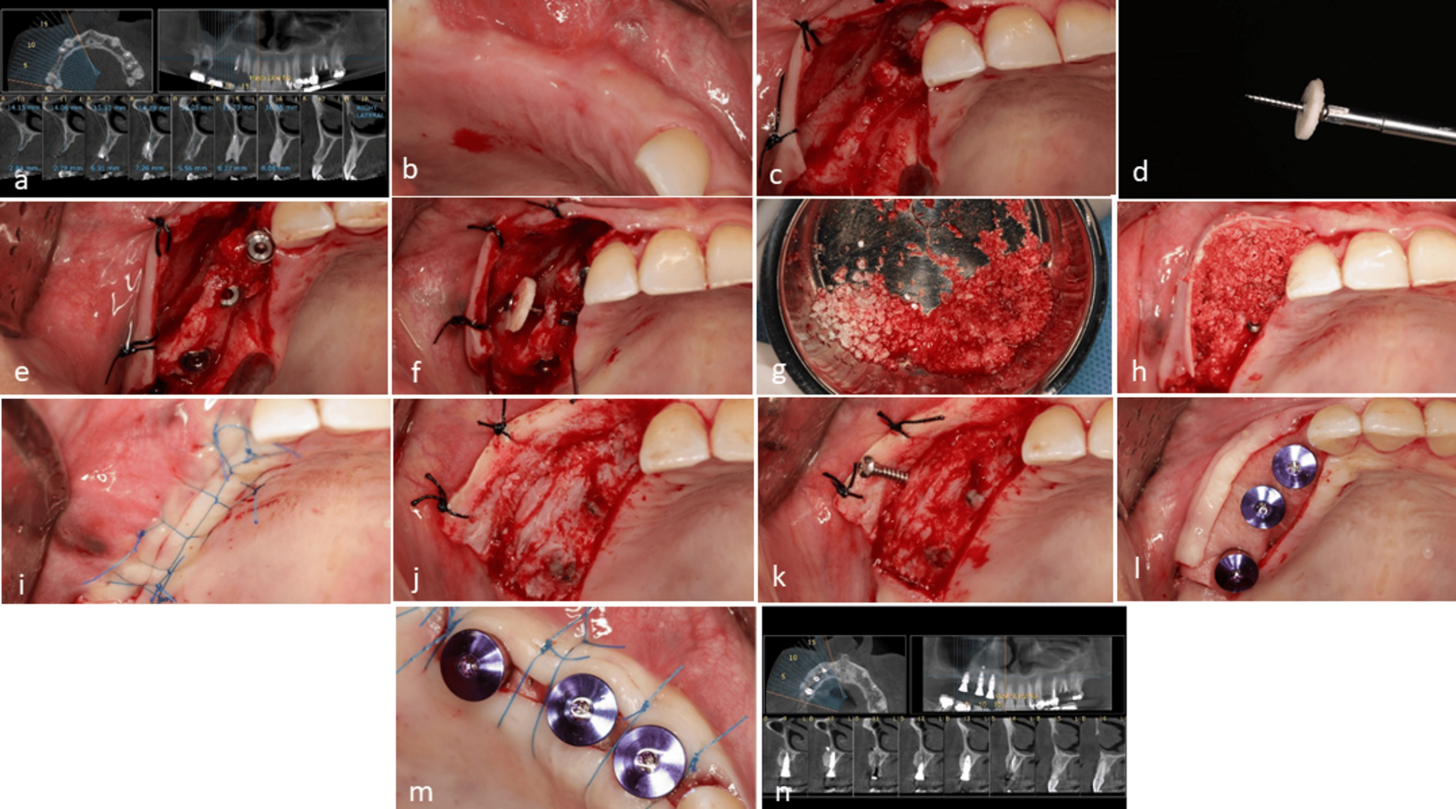
Case 1. (a) Pretreatment evaluation utilizing cone-beam computed tomography (CBCT) highlights the presence of non-restorable roots. (b) Clinical assessment was conducted after the designated healing period following the root extraction. (c) Observations indicate a bone defect accompanied by insufficient bone width. (d) Preparation of the cortical disc and screw assembly was conducted extraorally. (e) Implants were subsequently placed in the appropriate prosthetic positions; however, notable bone resorption in the canine site and inadequate contour in the posterior areas were observed. (f) Securing the screw and disc assembly adjacent to the central implant is essential to prevent soft tissue collapse. (g) Combination of allograft, autogenous chips, and xenograft materials. (h) A mixture of allo-xeno and autogenous material was implemented in the canine area, while the cortical disc was filled with an allo-autogen mixture, excluding xenograft. (i) Achieving primary closure during the initial surgical session. (j) After five months post-healing, the re-entry surgery was performed, and it was observed that the area lacking a cortical disc in the canine region experienced notable collapse. (k) Removal of the screw was conducted without detachment of the cortical disc. (l) Implant uncovering was conducted, incorporating an acellular dermal matrix to enhance soft tissue height. (m) Final suturing was performed. (n) CBCT following the re-entry appointment indicated adequate bone width over the implants, both in the presence and absence of the cortical disc.

A 37-year-old, non-smoking female was referred with non-restorable roots of the canine, first premolar, second molar, and third molar (Figure 2a). Following the extraction of these teeth, early treatment was scheduled for two months post-extraction (Figure 2b). Due to the observed level of bone resorption and destruction in the canine area, it was necessary to undertake bone reconstruction to facilitate the subsequent treatment. Following a thorough explanation of the reconstruction technique and obtaining the patient’s consent, a recommendation was made for administering amoxicillin 500 mg the day before surgery. On surgery day, a 0.2% chlorhexidine mouthwash (Iran Najo Chlorhexidine 0.2% Mouthwash, 250 mL; Iran) was used for one minute. A conventional flap was meticulously prepared using a mid-crestal incision. The clinical examination revealed significant destruction resulting from the canine extraction and horizontal resorption observed in the premolar and molar sites (Figure 2c). Despite this, the presence of the remaining bone allowed for the placement of three prosthetic-driven implants. However, the buccal surface bone did not have enough thickness to cover the implant surface during the remodeling process (Figure 2e). Therefore, the necessity for bone regeneration was evident. Following implant placement, the most significant loss was noted at the canine site. One cortical disc was prepared according to the established protocols. A screw (1.6 mm) was inserted into the disc (Figure 2d). The bone bed was adequately prepared to facilitate the insertion of the screw. To ensure optimal engagement between the screw and the bone bed, it is advisable to utilize a bi-cortical engagement technique. This approach effectively uses both the buccal and palatal cortices to achieve optimal stability of the cortical disc (Figure 2f).

After securing the cortical disc, the gap between the disc and the bone bed was filled with small-sized mineral allograft powder (FDBA, 500-1000 µm; Iranian Tissue Product Company). A xenograft membrane (Tutogen Medical GmbH; Germany) was employed to cover the assembly of the material and disc (Figures 2g, 2h). The surgical flap was secured with an internal horizontal mattress suture (Nylon 5-0, Nasj Tebb Keyhan; Iran) (Figure 2i).

Suture removal was performed 10 days after the surgery. The healing was uneventful. The re-entry procedure was performed five months after the initial surgery to remove the screw while preserving the integrity of the cortical disc (Figures 2j, 2k). In comparison, the regenerated bone demonstrated substantial coverage over the implants, indicating successful integration of the allograft cortical disc. To enhance the soft tissue condition, an acellular dermal matrix (thickness: 1.2-1.8 mm; Iranian Tissue Product Company) was used in conjunction with the implant uncover (Figures 2l, 2m). The comparison of radiographs taken before the extraction of the remaining roots and after the implants uncovering, particularly in the axial view, revealed a significant amount of new bone formation (Figure 2n).

Case 2

**Figure 3:**
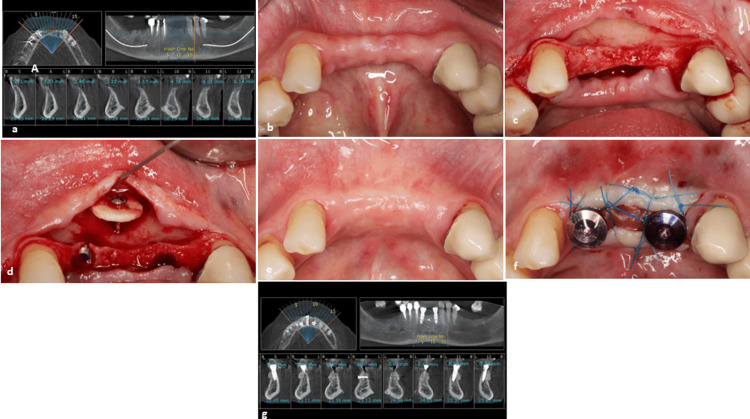
Case 2. (a) The pretreatment cone-beam computed tomography (CBCT) reveals an inadequate bone width in the anterior mandible. (b) A clinical assessment shows distortion of the alveolar ridge in the anterior region of the mandible. (c) A bone defect was identified following flap elevation. (d) Implants were placed, and a cortical disc with a screw assembly was attached in the inter-implant space to achieve an appropriate contour and enhance bone thickness. (e) A favorable bone contour developed five months post-surgery, as evidenced when compared to the initial clinical view (b). (f) Implants were uncovered, and an acellular dermal matrix was used to augment soft tissue height. (g) The follow-up CBCT indicates adequate bone width between the two implants and above the fixtures. It is important to note that the hidden screw could not be removed in this case.

A 63-year-old, non-smoking female was referred with reduced bone width in the anterior mandible. Pretreatment cone-beam computed tomography (CBCT) examinations confirmed the diminished bone width in this region, while clinical evaluation revealed an alveolar ridge deformity. Dental implants were placed in the desired locations, and a cortical disc was utilized to facilitate horizontal bone reconstruction. Following a five-month healing period, a satisfactory bone ridge contour was achieved, distinctly improved compared to the initial assessment. During the second surgical stage, the implants were exposed, and an acellular dermal matrix was employed to enhance the soft tissue thickness (Figure 3).

Case 3

**Figure 4:**
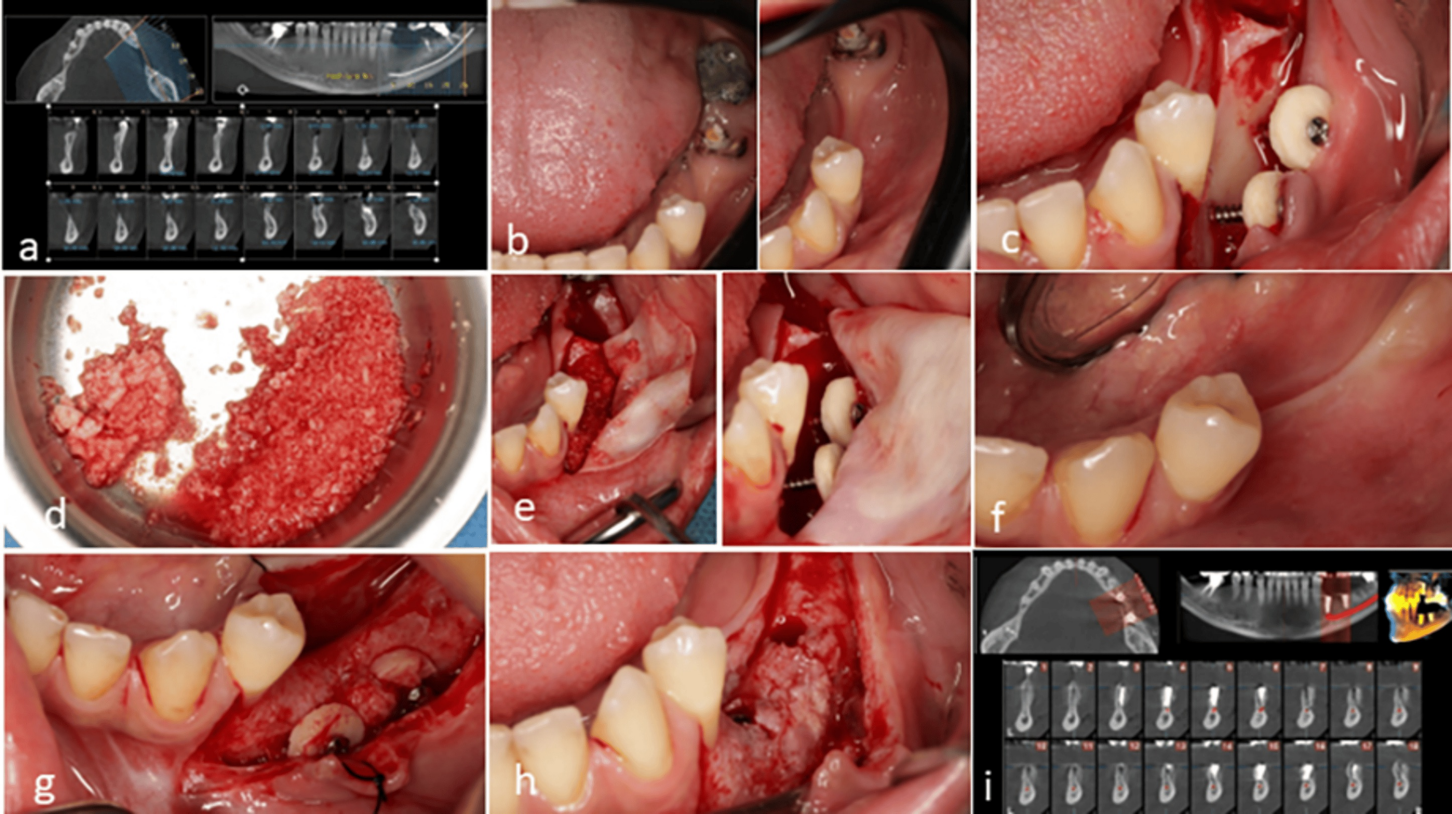
Case 3. (a) The cone-beam computed tomography (CBCT) analysis revealed severe atrophy in the posterior mandible. (b) A clinical evaluation of the edentulous site indicated the necessity for removing tooth roots. (c) Following the extraction, simultaneous implant placement was not feasible. Consequently, two cortical discs were utilized for bone augmentation. (d) A mixture of autogenous bone chips and allograft material was employed to fill the gap beneath the cortical discs. (e) The membrane was secured and the gap was filled with allograft and autogenous chips. (f) Clinical view after five months post-surgery. (g) A re-entry procedure confirmed adequate bone width beneath the cortical discs. (h) Implants were subsequently placed, demonstrating sufficient bone support on the buccal surface of the fixtures. (i) CBCT following the treatment showed satisfactory bone volume surrounding the implants.

A 36-year-old, non-smoking female was referred with significant bone loss in the posterior mandible. CBCT revealed a considerable bone deficiency in this area, which precluded the implant placement due to the narrow bone width. Following tooth extraction, a surgical approach was assumed utilizing two cortical discs along with a combination of autogenous bone and allograft material for bone regeneration. Five months after surgery, adequate bone formation was noted beneath the discs, allowing for successful implant placement (Figure 4).

Case 4

**Figure 5:**
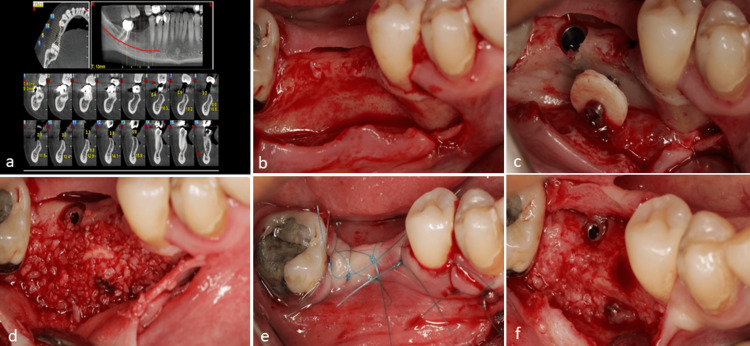
Case 4. (a) Presence of bone loss in the posterior mandible. (b) Clinical view of the bone defect. (c) Implant insertion and concurrent placement of the cortical disc and screw between the implants to enhance bone width. (d) Filling the gap beneath the cortical disc and the surrounding areas with a composite of allograft and autogenous chips. (e) Suturing. (f) A follow-up procedure was conducted five months post-treatment, demonstrating adequate bone width beneath the cortical disc and the adjacent areas.

A healthy 36-year-old female with bone loss in the lower jaw required horizontal bone reconstruction. During surgery, implants were placed, and a cortical disc was secured to enhance bone width. The area around the disc was filled with allograft and autogenous bone chips to promote new bone formation. A five-month follow-up showed a significant increase in the bone width in the surgical area (Figure 5).

Case 5

**Figure 6:**
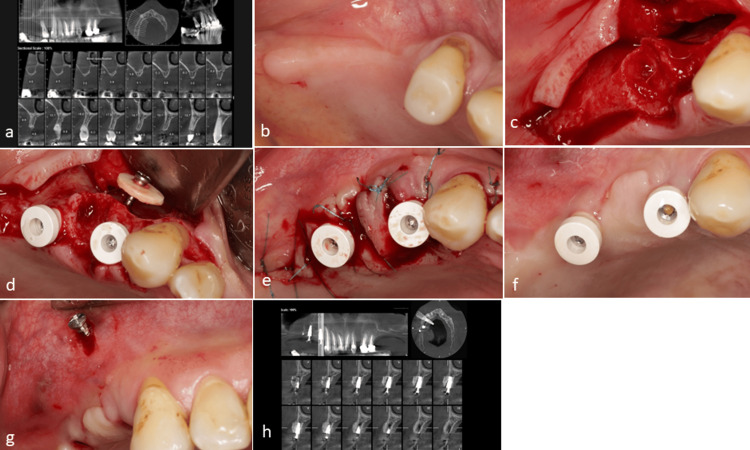
Case 5. (a) Preoperative cone-beam computed tomography (CBCT). (b) Clinical assessment conducted before the surgery. (c) A defect resulting from the first premolar extraction was observed after flap elevation, alongside significant horizontal bone resorption adjacent to the distal of the canine. (d) The cortical disc and screw assembly were installed in the distal portion of the first implant, ensuring adequate coverage for the defect at the distal of the canine. (e) A section of the palatal soft tissue was sutured and repositioned to facilitate defect closure between the two implants. (f) Healing was observed after five months, with adequate attachment tissue following the repositioning of the palatal flap. (g) The screw was removed without the need for flap elevation. (h) CBCT indicates sufficient bone in the area of the cortical disc and the surrounding region.

A 36-year-old, non-smoking male patient underwent a surgical procedure for the reconstruction of an edentulous area in the maxilla. After flap elevation, a significant horizontal bone defect was identified at the distal of the canine and first premolar. To address this defect, a cortical disc was placed in the distal portion of the first implant to ensure adequate coverage of the resorption site. Significant advancements were observed in tissue healing and soft tissue attachment after a five-month healing period. Additionally, CBCT validated the presence of sufficient bone formation in the reconstructed area (Figure 6).

Case 6

**Figure 7:**
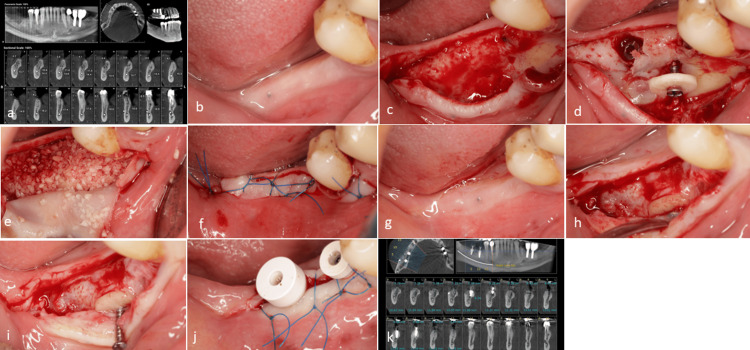
Case 6. (a) Preoperative cone-beam computed tomography (CBCT). (b) The clinical assessment before surgery revealed a narrow ridge. (c) Flap elevation confirmed the presence of alveolar bone resorption following tooth extraction; however, sufficient bone volume was noted to accommodate the implant placement. (d) A cortical disc was prepared to support the soft tissue before applying particulate bone materials. The screw was securely tightened into the cortical disc outside of the oral cavity and placed in the proper position. (e) The gap was filled with allograft pure cortical bone particulate. (f) A bilayer suturing technique was employed. (g) After five months, healing was uneventful, as evidenced by clinical observations. (h) Upon reflection of the flap, new bone formation beneath the cortical disc was observed. (i) The removal of the screw was performed successfully, with the cortical disc remaining in place due to adequate integration with the newly formed bone. (j) The final outcome after the tightening of the gingival former. (k) Postoperative CBCT indicates sufficient bone in the area of the cortical disc.

A healthy, non-smoking, 53-year-old male patient with a narrow alveolar ridge was referred for implant treatment. Following flap elevation, significant bone loss resulting from a previous tooth extraction was observed. After the placement of the implant to provide support for the soft tissue, a cortical disc was prepared and securely fixed in position. The space beneath the disc was then filled with pure cortical bone allograft particulate. The healing process progressed smoothly over five months. Upon re-entry, adequate bone formation had occurred beneath the disc, and the healing abutment was also tightened (Figure 7).

**Figure 8:**
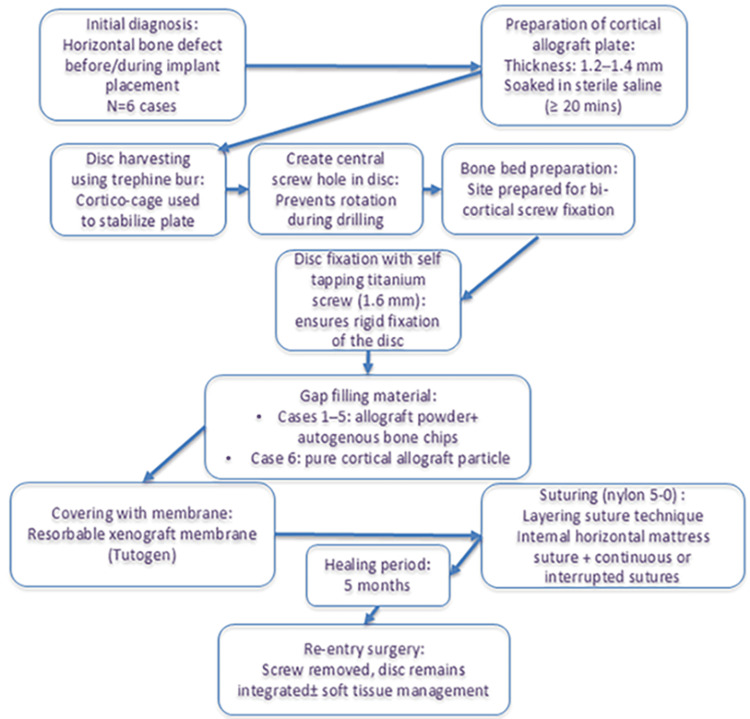
Flowchart depicting the single-screw cortical disc technique.

In five out of six cases, the cortical disc was simultaneously applied during implant placement. Only in Case 3, implant insertion was postponed and conducted in a second-stage surgery five months after bone regeneration (Figure 4). During the implant placement in Case 3, sufficient resistance was encountered during drilling, suggesting adequate bone density at the regenerated site. No postoperative complications such as infection, dehiscence, or disc mobility were observed in any of the six patients. All cases experienced a typical range of postoperative swelling and discomfort, which resolved within a few days. Cortical disc integration was achieved in all cases except Case 3, in which partial mobility of the disc was noted during the re-entry procedure. Despite this, there was adequate bone support around the implants, and no GBR was required after the healing period. According to the radiologist’s report, the average crestal bone width changes increased in all cases with available data. Specifically, in Case 1, it increased from 2.84 mm to 6.30 mm; in Case 2, from 3.22 mm to 6.10 mm; in Case 3, from 2.05 mm to 7.10 mm at the mesial plate site and from 1.74 mm to 7.50 mm at the distal plate site; in Case 5, from 3.70 mm to 6.80 mm; and in Case 6, from 4.40 mm to 6.43 mm. Postoperative CBCT imaging was not available for Case 4. The application of the technique mentioned above is illustrated through the flowchart depicted in Figure 8.

## Discussion

Adherence to the pass principles enhances the predictability of bone regeneration [10]. A fundamental aspect of new bone formation is maintaining or creating an optimal space between the periosteum and the bone bed. It is important to note that applying the GBR in extraosseous and large defects increases the risk of structural collapse [18]. Therefore, in such cases, the employment of tents or non-absorbable membranes becomes essential [19,20]. The shell technique is an effective method for reconstructing large extraosseous defects, which are particularly susceptible to collapse. This method is preferred for its capacity to improve space stability [14]. Numerous studies have been conducted on this method, all of which utilize a minimum of two screws to secure the bone plates [14]. This approach may necessitate a higher level of surgical expertise and increase the time required for the procedure, thereby elevating the complexity associated with bone regeneration treatment.

In the classic shell technique, the insertion of the screw into both the plate and the bone bed is conducted simultaneously and intraorally. To enhance the precision of alignment between the screw entry point in the cortical plate and the corresponding point in the bone defect, it is often necessary to perform simultaneous drilling of both the cortical plate and the bone intraorally. This study presents a modified approach to this technique that incorporates allograft plates as cortical discs. This method not only creates a space and facilitates the surgical process but also allows for the fixation of the plate using a single screw from outside the oral cavity.

Therefore, two key modifications, namely, the circular design of the cortical plate and the attachment of the screw to the plate outside the oral cavity, contribute to the simplification of the surgical technique. Moreover, the circular configuration of the cortical plate enables simultaneous fixation at the defect site during screw insertion, without interference from adjacent anatomical prominences. This approach significantly reduces treatment time and minimizes the requirement for supplementary equipment. As mentioned earlier, the bone plates employed in the shell technique can be sourced from various origins. Autogenous bone blocks [21] are considered the gold standard among graft materials, owing to their osteogenic, osteoinductive, and osteoconductive properties [22]. Nonetheless, this approach presents certain limitations and challenges. These include concerns related to the limited availability of bone, potential donor site pain and infection, as well as the possibility of sensory neuropathy [23].

The utilization of allograft [14,24] and xenograft [15] rigid barriers presents a viable alternative to autologous cortical blocks. This approach not only achieves comparable clinical outcomes to those of autologous grafts [13] but also mitigates the necessity for bone harvesting from the patient. Consequently, this method reduces pain, postoperative morbidity, procedural duration, and overall surgical complexity [14,25]. A comparative analysis of allograft and autologous block resorption indicates that the resorption rate for autologous blocks (43.7%) exceeds that of allografts (31%) [26,27]. This higher resorption rate contributes to enhanced stability and longevity of the created space over time. Consequently, the utilization of allograft cortical plates has experienced a significant increase in recent practice for bone reconstruction in both horizontal and vertical dimensions.

Our study aligns with the findings of the Tunkel split mouse investigation, particularly regarding bone gain observed in both horizontal and vertical dimensions. This study utilized both autogenous and allograft plates. The results indicate that bone gain and resorption can occur in both dimensions, with no significant differences between the two groups. Notably, despite the extended regeneration period associated with allogeneic bone plates, no undesirable bone loss was recorded in the long term following the procedure [14].

When utilizing the shell technique with conventional allograft plates, which are typically prefabricated in a rectangular configuration, it is often necessary to modify the plate to accommodate the specific dimensions of the defect [14,24]. The implementation of a cortical disc with a single screw facilitates this process, thereby obviating the requirement for additional re-shaping before or after fixation.

Any movement of the cortical plates during the healing period poses a risk of treatment failure [28]. Consequently, the conventional fixation method typically employs a minimum of two screws; in some instances, additional screws may be utilized to reduce further lateral movement. However, it is essential to note that increasing the number of screws not only extends the required time for the procedure but also increases the possibility of cracking in the cortical plate.

The simplified introduced technique utilized customized plates with a thickness ranging from 1.2 to 1.4 mm to ensure optimal engagement between the screw and the plate. Clinical outcomes observed following the established healing period indicate that both the screw and the cortical disc maintained their positions securely as an integrated unit.

To ensure optimal screw integration, it is essential to select the appropriate thickness of the cortical disc, as well as a screw diameter (at least 1.4 mm). By considering these factors, concerns regarding screw integration can be significantly alleviated. Furthermore, the circular design of the modified plates minimizes the presence of sharp edges, thereby reducing the risk of wound exposure.

It is advisable to select a screw length that facilitates bi-cortical fixation across the entire width of the defect to ensure optimal stability of the screw-disc assembly. The number of cortical discs utilized should be determined based on the size of the defect. Given the straightforward nature of this technique, it is feasible to employ multiple cortical discs close to one another.

It is crucial to prioritize filling the gap beneath the plate with autogenous chips in all shell techniques, whether using autogenous or non-autogenous plates [14,15]. However, we observed that, in most cases analyzed, small-sized cortico-cancellous allograft powder (FDBA, 500-1,000 µm; Iranian Tissue Product Company) was the predominant material used. In only one case (Case 6), the existing gap was filled exclusively with pure cortical allograft particles, which did not lead to any noticeable difference in the clinical outcome. Furthermore, a waiting period of four to five months was consistently observed across all cases. Although incorporating autogenous chips may potentially diminish the waiting period, the outcomes associated with allograft powder are anticipated to be comparable to those achieved with autogenous chips [29].

The optimal thickness of the cortical plate in conventional shell techniques is generally 1 mm or less. A reduction in this thickness appears to correlate with an increased likelihood of the plate successfully integrating with the new bone. However, considering that the thickness selected for achieving maximum stability in cortical discs exceeds 1 mm, there exists a risk of disc separation during the re-entry phase associated with screw removal. Using these plates is primarily intended to create adequate space so that it does not impact the amount of new bone formation. While increasing the thickness of the plate can enhance the stability of the disc-screw assembly, it is essential to note that excessive thickness may lead to an increased rate of plate resorption (Figure 4g) [16].

While the benefits outlined above are significant, it is essential to recognize that any mobility following cortical disc fixation is considered unacceptable and can lead to treatment failure. Unlike traditional techniques, there is no option to incorporate additional screws to enhance cortical disc stability. Therefore, if the cortical disc does not achieve adequate stability, it must be repositioned to ensure optimal contact between the screw and the bone bed.

It is advised that for low-density bone, the diameter of the hole prepared in the bone bed be slightly smaller than the diameter of the screw intended for use. Conversely, in high-density bone such as D1, it is often essential to prepare a bone hole that is proportional to the screw diameter.

This method has two main limitations. First, compromised soft tissue may lead to early exposure of the cortical disc. Second, insufficient bone width in the defective site may compromise the screw engagement to the bed.

While all methods involving a rigid barrier (block grafts, expanded polytetrafluoroethylene, dense polytetrafluoroethylene, titanium mesh) demonstrate superior effectiveness in creating space compared to resorbable membranes, the success of these interventions depends on adequate soft tissue coverage. Therefore, it is prudent to exercise caution when selecting this type of barrier. Any exposure related to cortical plates may jeopardize the success of bone regeneration treatments. In all cases presented in this study, the soft tissue at the surgical site was keratinized and exhibited a thickness greater than 2 mm. As a result, soft tissue healing proceeded uneventfully throughout the postoperative period.

This case series aimed to illustrate the clinical applicability and effectiveness of the proposed technique in both maxillary and mandibular sites. Given the structural role of cortical plates as a rigid barrier, the technique may be particularly advantageous in managing extraosseous defects.

Despite the promising outcomes observed in the presented cases, the limited sample size and the current study design represent notable limitations. It is recommended that future research be conducted as randomized controlled trials, preferably using a split-mouth design, to allow for a more robust comparison between this modified technique and the conventional shell technique.

## Conclusions

The findings of this case series demonstrate that implementing the single-screw cortical disc technique can serve as a promising technique in horizontal bone augmentation. Reduced surgical time and increased facility for utilization of the cortical plates may be significant advantages of this technique. Moreover, using a single screw provides a notable benefit in securing the cortical disc and connecting it to the disc outside the patient’s oral cavity.
